# Amharic oral health tools for refugees: a hybrid review of OHIP-14 and WHO adaptations

**DOI:** 10.1186/s12903-026-07801-0

**Published:** 2026-02-11

**Authors:** Betelehem Ketema, Karen Lansdown, Zeina Al Naasan, Heuiwon Han, Julie Trafford

**Affiliations:** 1 School of Community and Public Health, Auckland University of Technology, Auckland, New Zealand; 2 Department of Oral Health, School of Acute and Primary Health, Auckland University of Technology, Auckland, New Zealand; 3 Sir John Walsh Research Institute, University of Otago, Dunedin, New Zealand

**Keywords:** Amharic language, Cross-cultural adaptation, OHIP-14, Oral health–related quality of life, Psychometric validation, Refugee oral health

## Abstract

**Background:**

Despite the growing need for culturally valid oral health tools in refugee populations, no validated Amharic-language versions of key instruments currently exist. This review synthesises how the OHIP-14 and WHO Oral Health Assessment tools have been adapted across linguistic and cultural contexts, with implications for Amharic-speaking Ethiopian refugees.

**Aim:**

To assess how OHIP-14 and WHO-OHAFT have been cross-culturally adapted and validated globally, and to identify gaps and equity implications for developing Amharic-language tools in refugee contexts.

**Methods:**

We conducted a hybrid systematic–narrative review of 21 studies, using structured database and grey-literature searches followed by descriptive mapping and thematic synthesis. Studies were charted by language, adaptation procedures, and psychometric properties (e.g., Cronbach’s α, intraclass correlation coefficients). Cross-cultural adaptation frameworks, such as those of Beaton et al. and WHO translation guidelines, guided the assessment of methodological and linguistic rigour across studies.

**Results:**

Three main themes emerged: [1] consistent psychometric strength across diverse cultural adaptations; [2] methodological variation and reporting gaps in cross-cultural validation; and [3] a complete absence of validated Amharic-language tools. While Cronbach’s α values ranged from 0.72 to 0.99 (mean = 0.88), many studies omitted essential adaptation steps. Refugee-specific oral health beliefs, such as spiritual interpretations of pain, are rarely integrated.

**Conclusion:**

This review highlights both strong potential and critical limitations in current cross-cultural oral health assessments. It emphasises the ethical and clinical needs for developing validated, culturally appropriate Amharic tools. Cross-cultural adaptation should be seen as a step towards linguistic justice and oral health equity for Amharic-speaking refugee and displaced populations.

## Introduction

Oral health is increasingly recognised as a critical component of overall well-being, influencing essential functions such as eating, speaking, and smiling, as well as social participation, self-esteem, and mental health [1]. Untreated conditions such as dental caries, periodontal disease, and oral infections can cause pain, impair daily activities, and substantially diminish quality of life [2]. Periodontal disease has also been linked to systemic conditions, including cardiovascular disease and diabetes, underlining the broader health consequences of unmet oral health needs [2, 3].

These challenges are amplified among refugee populations, who face unique barriers to maintaining oral health during and after displacement [1, 4]. Food insecurity, limited oral health literacy, and unfamiliarity with health systems in resettlement countries are common structural obstacles [1, 4–6]. Among Ethiopian communities, traditional practices such as using chewing sticks (mefakia) and consuming diets high in fibre and low in sugar have historically supported good oral health by promoting saliva production and reducing microbial load [7, 8]. However, these protective behaviours often decrease during displacement from home countries, as access to oral hygiene tools, nutrient-rich foods, and dental care diminishes [4, 8]. Malnutrition, common in refugee settings, can further impair oral and systemic health via nutrient deficiencies [1]. Trauma-related conditions such as anxiety, depression, and bruxism may further compound vulnerability to poor oral health in resettlement contexts [9–11]. Local evidence from Aotearoa New Zealand echoes these patterns, suggesting high levels of unmet oral health need and persistent structural barriers to care among resettled refugees [5].

Despite these growing concerns, Amharic-speaking refugee populations remain notably underrepresented in oral health research and surveillance systems [1, 9]. Amharic is spoken by a large proportion of Ethiopia’s population and is the dominant language among Ethiopian refugees [1, 10]. In Aotearoa New Zealand, census data identifies over 2,100 people as Ethiopian by ethnicity [11], yet no national oral health studies have systematically examined outcomes or quality of life within this group. This invisibility raises concerns about equity and the recognition of Amharic-speaking refugees’ experiences in policy and practice.

Critically, there are currently no validated Amharic-language versions of key oral health tools such as the OHIP-14 (Oral Health Impact Profile–14) or WHO-OHAFT (World Health Organization Oral Health Assessment Forms (WHO Oral Health Surveys). These tools respectively measure subjective oral health–related quality of life (OHRQoL) and clinical oral health indicators [12, 13]. While widely used globally, their effective deployment in refugee settings requires rigorous cross-cultural adaptation to ensure semantic and conceptual relevance [14]. Cross-cultural adaptation frameworks emphasise that linguistic equivalence alone is insufficient; instruments must also capture culturally specific meanings, norms, and experiences [14, 15].

Without such adaptation, tools may fail to capture culturally grounded beliefs, traditional practices, or symptom expression patterns, risking both misdiagnosis and misrepresentation [14, 16]. This absence reflects broader gaps in oral health equity and linguistic inclusion, where minoritised refugee communities are overlooked in measurement science [1]. From an equity perspective, cross-cultural adaptation is not only technical but also a matter of linguistic justice, ensuring that Amharic-speaking refugees are able to articulate their oral health experiences in their own language and on their own terms [1]. This hybrid systematic–narrative review, therefore, aims to [1] synthesise how the OHIP-14 and WHO-OHAFT have been cross-culturally adapted and validated across diverse linguistic and cultural contexts, and [2] explore the implications of this evidence for developing validated Amharic-language oral health tools for refugee populations.

## Method

### Clarifying the purpose and research questions

This review aimed to explore how OHRQoL is assessed in refugee populations, focusing on Amharic-speaking communities and the adaptation of the OHIP-14 and WHO-OHAFT. The following research questions guided the review:

- RQ1: How have the OHIP-14 and WHO-OHAFT been applied in oral health research involving refugee or ethnolinguistically diverse groups, particularly in Amharic-speaking communities?
- RQ2: What methods have been used to adapt, translate, and validate these tools across diverse linguistic and cultural settings?

### Justifying the review type

This review used a hybrid systematic-narrative approach guided by Turnbull et al.’s (2023) six-step framework [17], which involves:

1. Clarifying the purpose and research questions.
2. Justifying the review type.
3. Searching and selecting literature.
4. Extracting and organising data.
5. Synthesising and interpreting findings.
6. Writing the review.

A hybrid design was chosen because it deliberately integrates two methodological streams:

- a structured, reproducible systematic component (searching, screening, extraction), and.
- an interpretive narrative component (contextual, cultural, and thematic analysis).

This combination provides both methodological rigour and the interpretive depth needed to explore sociocultural, linguistic, and psychometric complexity [18–20]. It is well-suited to cross-cultural research, where adaptation processes vary and methodological heterogeneity is common [14, 16, 19].

Given the diversity of qualitative and quantitative approaches in OHRQoL, refugee health, and instrument-validation studies [4, 13, 21], a hybrid design enabled structured search procedures to be aligned with contextual interpretation while maintaining transparency [17, 22]. This was essential for identifying methodological gaps and cultural considerations in tool adaptation, especially in settings lacking validated Amharic-language instruments [14, 16].

### Hybrid systematic–narrative integration

This review followed a hybrid systematic–narrative approach as outlined by Turnbull et al. [17, 22]. The systematic stream comprised the structured database and grey-literature searches, application of the PICO-based (Population, Intervention/Indicator, Comparison, Outcome) inclusion and exclusion criteria, and extraction of methodological and psychometric variables into the synthesis matrix. The search concepts and key terms used across databases are summarised in Table 1, and the inclusion and exclusion criteria are presented in Table 2. The flow of records through identification, screening, eligibility assessment, and final inclusion is depicted in the PRISMA 2020 diagram (Fig. 2).

**Table 1:** Search concepts and key terms used in the literature search strategy

Search concepts	Key words
Tool names	“Oral Health Impact Profile” OR “OHIP-14”“World Health Organisation Oral Health Assessment” OR “WHO Oral Health Survey”
Adaptations Methods	“cross-cultural adaptation” OR “translation” OR “validation”
Populations	“refugee” OR “migrant” OR “ethnic minority” OR “Amharic” OR “Ethiopian”
Domain Focus	“Oral health” OR “oral health-related quality of life” OR “OHRQoL”

**Table 2:** Inclusion and exclusion criteria

PICO	Inclusion	Exclusion
Population	- Refugee, migrant, or ethnic minority populations (including displaced or resettled groups) - Ethiopian or Amharic-speaking groups	- Studies limited to non-refugee or general populations
Interest	- Oral health status - Quality of life related to oral health (OHRQoL) - Utilisation of validated OHIP-14 or WHO-OHAF instruments for assessing oral health-related quality of life (OHRQoL)	- Studies focusing only on dental treatment outcomes (e.g. fillings, scaling) without quality-of-life or tool-based assessment
Construct	- Cross-cultural validation or adaptation of OHIP-14 or WHO tools - Studies reporting translation, cultural adaptation, or psychometric evaluation of oral health measurement tools	- Studies using tools without documented validation, adaptation, or translation processes - No methodological detail on tool adaptation or translation
Other Aspects	- Peer-reviewed articles - Quantitative, qualitative, or mixed methods designs - Published between 2000 and 2024 - English language or translated text	- Editorial, commentaries, or opinion pieces - Non-English articles without translation - Published before 2000

The narrative stream involved interpretive synthesis of cultural, linguistic, contextual, and refugee-specific themes derived from the mapped data [20].

**Fig. 1:**
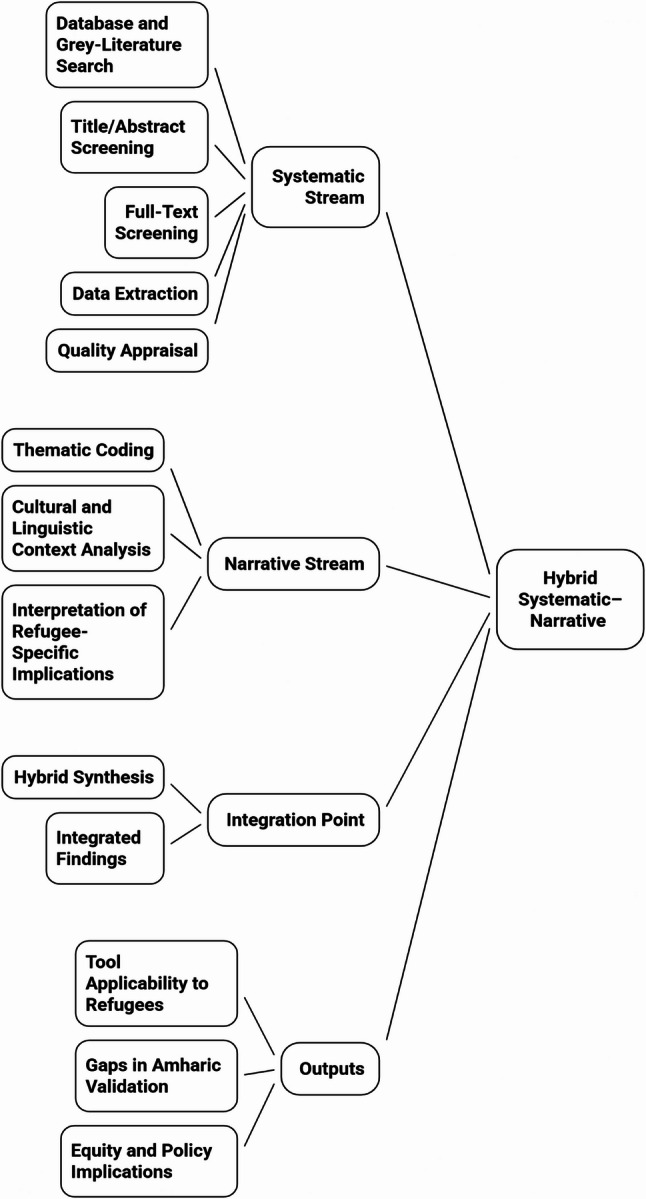
Hybrid systematic–narrative review integration

A methodological integration flowchart (Fig. 1) illustrates how the systematic (quantitative mapping) and narrative (thematic) streams were linked within the hybrid review design. Cross-cultural and multilingual adaptation frameworks (e.g., Beaton et al., Sousa & Rojjanasrirat) informed the classification of adaptation procedures and psychometric indicators within both streams, supporting consistent comparison of tool adaptations across languages and settings [22].

Flowchart illustrating the parallel systematic (quantitative mapping) and narrative (thematic synthesis) streams and their integration to generate findings on tool applicability, gaps in Amharic validation, and equity implications [17].

### Searching and selecting literature

Studies were selected using structured inclusion and exclusion criteria based on the PICO (Population, Intervention, Comparison, Outcome) framework [23].

To ensure comprehensive coverage across indexing systems, search terms were organised into concept groups that captured tool names, adaptation methods, population descriptors, and oral-health domains.

A summary of these concepts and key terms is presented in Table 1.

Eligible studies focused on refugee, migrant, or ethnolinguistically diverse populations, including but not limited to Amharic-speaking Ethiopian groups [4, 6]. General population studies were included when they reported translation, cross-cultural adaptation, or psychometric validation of OHIP-14 or WHO-OHAFT in any linguistic or cultural setting, provided that these findings contributed methodological relevance to Amharic-language or refugee contexts [14, 16, 24]. Because only one Amharic-specific study was identified, broader linguistic and cultural adaptations were included to allow meaningful comparison and to identify gaps relevant to Amharic-language tool development [17–19].

A summary of all inclusion and exclusion criteria is presented in Table 2.

**Fig. 2:**
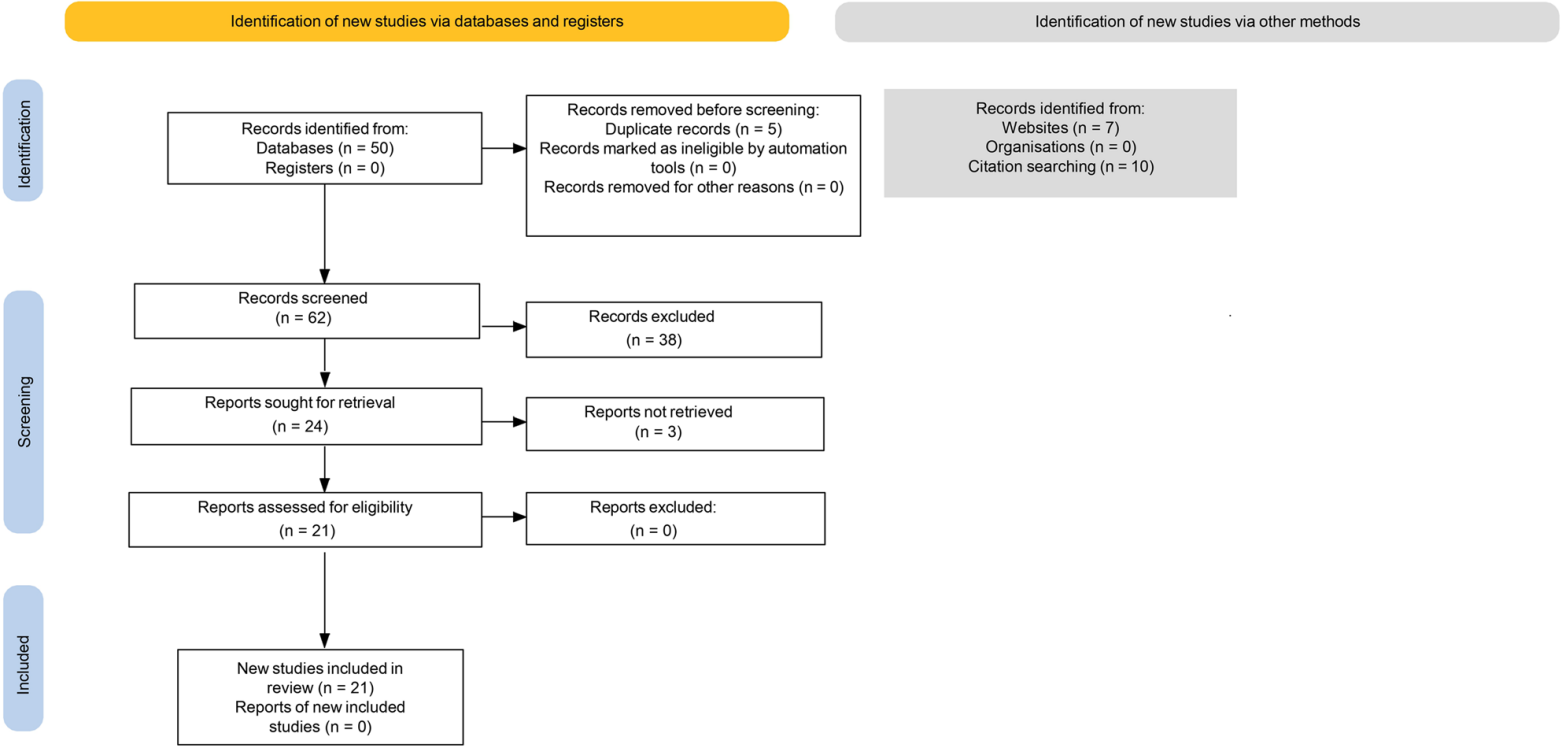
PRISMA 2020 flow diagram

After completing the database searches (PubMed, Scopus, ScienceDirect, and Google Scholar) and identifying additional records through manual reference checking and citation tracking, all retrieved records were compiled into a single dataset and de-duplicated. Database searches identified 50 records; together with grey-literature and citation-tracked sources, this resulted in 67 records in total. After removal of 5 duplicates, 62 unique records remained for screening (Fig. 2). Screening was guided throughout by the predefined PICO-based inclusion and exclusion criteria [19].

Title and abstract screening was conducted by one reviewer (BK), applying these criteria to assess relevance to OHIP-14 or WHO-OHAFT use, translation, validation, or cross-cultural adaptation within refugee, migrant, or ethnolinguistically diverse populations. The screening criteria and decision-making framework were co-developed with the wider research team to ensure methodological rigour and consistency. At this stage, 38 records were excluded because they did not reference the tools of interest, did not involve translation or adaptation processes, were unrelated to refugee, migrant, or linguistically diverse populations, or clearly duplicated data reported elsewhere.

Full texts were sought for the remaining 24 records; three could not be retrieved. The 21 accessible full-text articles were assessed for eligibility independently by two reviewers (BK and HH), using the inclusion and exclusion criteria to consider psychometric reporting, cultural-adaptation processes, and alignment with the research questions. The full-text screening procedures and decision framework were reviewed and validated by the wider research team to ensure consistency and methodological rigour. Discrepancies in screening decisions were resolved through discussion to minimise selection bias and enhance reliability.

The diagram illustrates records identified, screened, assessed for eligibility, and included in the review. Database searches identified 50 records, and additional sources identified 17 records through websites and citation tracking. After removing duplicates (*n* = 5), 62 records were screened, 24 full texts were assessed, 3 could not be retrieved, and 21 studies were included.

### Extracting and organising data

After full-text eligibility was confirmed, data from each included study were extracted into a structured synthesis matrix. This matrix was co-developed with the wider research team to ensure comprehensive coverage of psychometric, methodological, and cultural-adaptation domains. One reviewer (BK) completed the initial extraction, and a second reviewer (HH) independently checked the entries, with discrepancies resolved through discussion to ensure accuracy and consistency.

Key methodological and contextual variables were extracted, including study design, population characteristics, cultural or linguistic context, translation or adaptation procedures, psychometric indicators (e.g., Cronbach’s alpha [Cronbach’s α], intraclass correlation coefficients [ICCs]), validity measures, and reported limitations. Adaptation procedures were coded with reference to established cross-cultural and multilingual adaptation frameworks (e.g., Beaton et al., Sousa & Rojjanasrirat) to enable consistent comparison of processes across languages and settings [14, 16]. These variables were entered into the synthesis matrix to enable structured comparison across studies. Psychometric properties were interpreted using the relevant components of Terwee et al.’s quality criteria, including internal consistency, test–retest reliability, and construct validity, providing a structured appraisal of measurement quality and risk of bias [24].

Of the 21 included studies, 14 focused on OHIP-14 adaptations, three examined WHO-OHAFT, and four provided additional psychometric or cultural-adaptation data relevant to the review. This structured extraction process ensured that all variables reported in the final synthesis were verifiable from the included studies. Rigour was strengthened through collaborative discussion within the research team, who reviewed extraction decisions, methodological classifications, and interpretation of adaptation procedures, in line with Turnbull et al.’s guidance on maintaining transparency and reducing single-reviewer bias in hybrid systematic–narrative reviews [17].

### Synthesising and interpreting findings

The final phase of this hybrid review involved synthesising the extracted data using a combined descriptive mapping and inductive thematic analysis approach [17, 20, 25]. The synthesis drew on the extracted variables, which included study design, population characteristics, cultural context, adaptation procedures, and psychometric indicators [17, 19].

In the descriptive mapping phase, key study attributes, such as country, instrument type, linguistic or cultural setting, and adaptation procedures, were summarised to provide contextual comparability across studies. This mapping highlighted variation in translation processes, expert involvement, pre-testing, and psychometric reporting, and allowed us to note whether studies referenced recognised cross-cultural adaptation frameworks [14, 16, 26–36].

The thematic synthesis was developed iteratively. Patterns related to methodological consistency, cultural adaptation practices, and psychometric strength were identified through repeated review of extracted data. Interpretations were refined collaboratively within the wider research team, strengthening transparency and reducing single-reviewer bias. This collaborative approach aligned with the hybrid design and enhanced the credibility of theme development without implying a formal qualitative coding protocol [25].

To support systematisation across diverse methodologies, methodological variables, adaptation steps, and psychometric indicators were compared across studies within the matrix. Integrating descriptive mapping with emerging thematic patterns allowed quantitative evidence (e.g., reliability coefficients) and qualitative insights (e.g., cultural adaptation processes) to be interpreted together, providing a holistic understanding of adaptation practices and gaps relevant to Amharic-language tool development [14, 16].

### Adaptation and validation findings

**Fig. 3:**
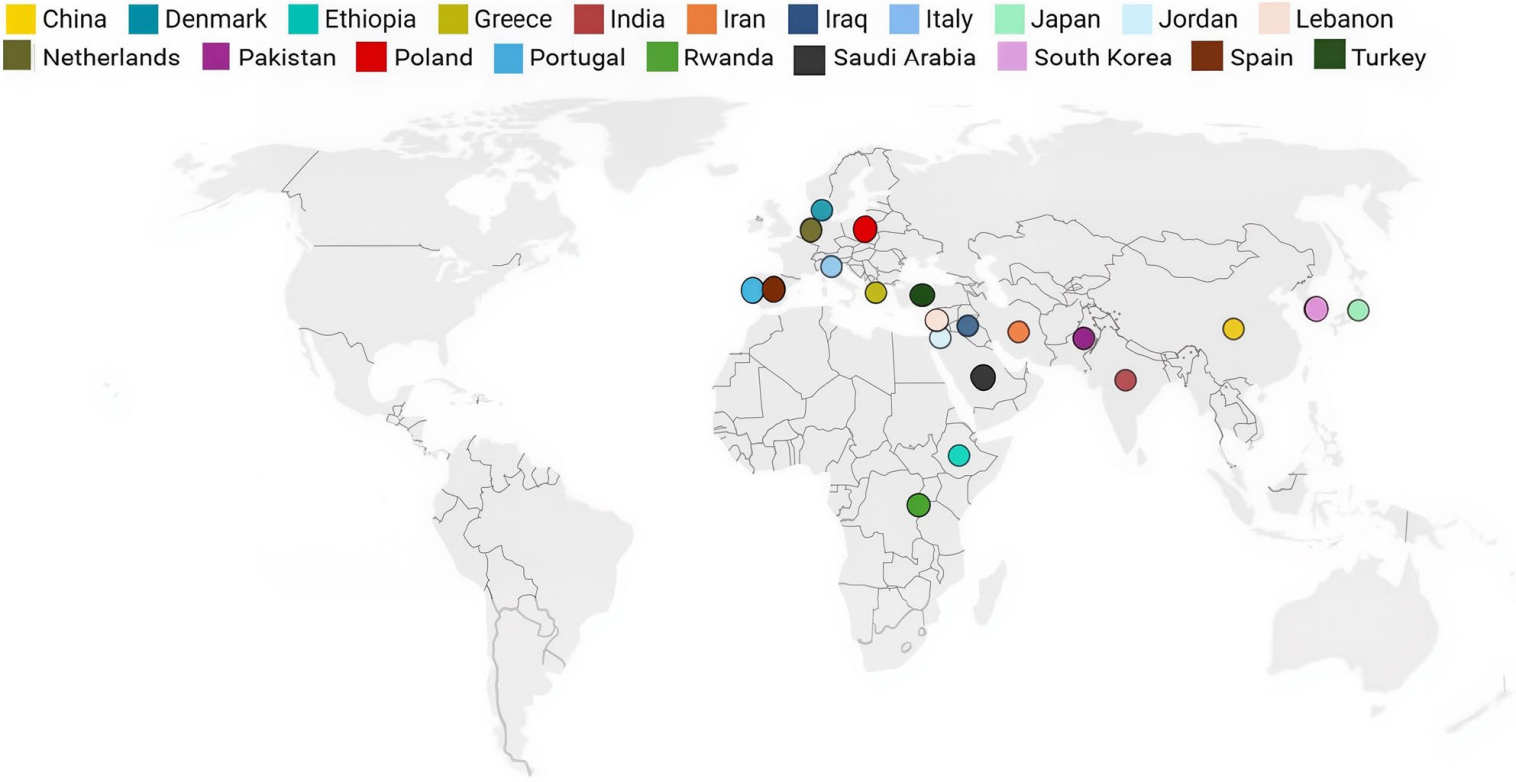
Geographic distribution of included studies validating OHIP-14 and WHO oral health tools

Twenty-one studies published between 2007 and 2022 were included in the final synthesis [7, 26–47]. These examined the cultural adaptation and psychometric validation of the OHIP-14, WHO-OHAFT, or OHAT (oral health assessment tools) across 20 countries and 21 languages. The studies covered populations in Asia, the Middle East, Europe, and Africa (Fig. 3), and included both general community samples and specific groups such as older adults [26, 39, 41, 48], adolescents [7, 38, 40], and individuals with intellectual or physical disabilities [28, 41]. None of the included studies explicitly involved refugees or recently resettled migrants; all were conducted in general community or clinic-based samples in the countries of interest.

Most studies used cross-sectional validation designs incorporating forward–backward translation, expert panel review, and pilot testing [27, 30, 36, 37, 39, 42]. Several also complemented survey administrations with clinical oral health examinations or structured interviews [27, 33, 34, 37, 42]. Reported psychometric outcomes indicated Cronbach’s α values ranging from 0.70 [43] to 0.97 [26], and most studies reported ICCs above 0.80 [27, 32, 36, 39]. Several studies also conducted factor analyses to confirm construct validity [31, 35, 40], and associations were observed with clinical indices such as periodontal status, Decayed, Missing, and Filled Teeth (DMFT) scores, and self-rated oral health [33, 37, 43, 45]. Four adaptations of Arabic-language tools were validated in Jordan [37], Lebanon [39], Saudi Arabia [38], and among Arabic-speaking populations in Iran/Iraq [31]. In India, validations were reported for both Hindi [46] and Gujarati [30]. In Africa, adaptations were reported in Rwanda [32] and Ethiopia [7], with Ethiopia providing a basic translation of WHO-OHAFT. Across Europe, validations were reported for versions in Dutch [36], Danish [29], Spanish [42], Portuguese [28], Polish [45], Greek [33], and Italian [41].

Among the included studies, one study focused on Amharic-speaking populations in Ethiopia, and it involved a basic translation of the WHO-OHAFT [7]. Participant involvement in translation processes (e.g., cognitive interviewing, focus groups) varied across studies, with several relying primarily on expert review [7, 27, 38]. Table 3 summarises the characteristics of the included studies, including settings, populations, instruments, and cultural adaptation processes.

**Table 3:** Summary of cross-cultural adaptation and psychometric properties of OHIP-14 and WHO-OHAFT

Author(s) and Year	Language of tool	Country	Population	Study Design/ Methodology	Instrument(s) Used	Key Features	Psychometric Outcome(s)	Domain/ Thematic Relevance
Al Habashneh et al. (2012) [37]	Arabic (Jordan)	Jordan	Adults (*n* = 400) aged 18–60 with varying periodontal disease severity	Cross-sectional validation study using clinical exams and OHIP-14 survey	OHIP14 (Arabic version)	Systematic translation and pilot use; clinical exam by trained dentists; subscale analysis across disease severity	Cronbach’s α = 0.89; significant associations with disease severity (multivariate analysis, *p* < 0.05)	OHRQoL assessment; cross-cultural validation in a clinical context; oral health disparities
Bae, Kim, Jung et al. (2007) [26]	Korean	South Korea	Elderly adults aged ≥56 (*n* = 1,098)	Cross-sectional validation study using forward–back translation, expert review, pilot testing, and test–retest reliability (3-month interval)	OHIPK (full version) & OHIP14K (Korean short form)	Forward–back translation, pilot testing, test–retest (3 months), interviewer-administered survey	OHIPK: Cronbach’s α = 0.97; ICC = 0.64. OHIP14K correlated strongly with full version (R² = 0.96)	OHRQoL measure validation among older Korean adults; established a culturally relevant short form
Balcı et al. (2017) [27]	Turkish	Turkey	Adults attending medical check-ups	Cross-sectional validation study incorporating interviews and periodontal records	OHIP14-TR (Turkish)	Forward/back translation with expert panel; pilot testing; tested comprehensibility (>95%)	Cronbach’s α = 0.76–0.91 across three subsamples ICC = 0.89; Spearman’s correlations with periodontal indices (*p* < 0.01)	Instrument adaptation; OHRQoL in general clinical settings
Bokhari and Quadri (2020) [38]	Arabic	Saudi Arabia (Jazan)	High school adolescents (*n* = 478, 16.3 ± 1.0 yrs)	Cross-sectional validation using pilot-tested A-OHAT	A-OHAT (Arabic WHO Child-OHAT)	Forward/back translation, piloted with 50 adolescents; administered by trained dental students	Cronbach’s α = 0.72; ICC = 0.89; no significant test–retest differences; convergent validity via Spearman rvalue > 0.50 (*p* < 0.05)	Child oral health assessment, tool validation in Arabic-speaking adolescents
Couto et al. (2018) [28]	Portuguese	Portugal	Adults with mild intellectual disabilities (*n* = 240)	Cross-sectional evaluation with clinical oral exams and interviews	OHIP14-MIDPT	Forward/back translation; pilot-tested; intraoral exam; cognitive interviewing	Cronbach’s α = 0.922; ICC = 0.999; item-total *r* = 0.529–0.718; inter-item *r* = 0.277–0.749; convergent & divergent validity supported (*r* = –0.545, *p* < 0.001)	Validation in special-needs population; psychometric rigour
Deshpande & Nawathe (2015) [46]	Hindi	India (Vadodara)	Adults at dental clinics (*n* = 102)	Cross-sectional translation & validation with pilot-testing & clinical exams	OHIP14 (Hindi)	Forward/back-translation, pilot tested on 48 participants, bilingual pre-test; used Likert scale & 1-month recall period	Cronbach’s α = 0.80; Pearson *r* = 0.963; no significant difference between English/Hindi versions (t = ns), corrected item-total *r* > 0.2; associations with OHIS scores	Cross-cultural validation; OHRQoL tool adaptation; linguistic equivalence
El Osta et al. (2012) [39]	Arabic	Lebanon (Beirut)	Older adults ≥65 yrs living independently (*n* =206)	Comparative cross-sectional study with structured interviews and clinical exam; cultural adaptation of OHIP14 & GOHAI; test–retest in subsample	Arabic OHIP14 & GOHAI	Expert review; pilot study with 87 elders; cultural rewording of selected items; trained interviewers; dual scoring (ADD & SC)	Cronbach’s α > 0.88 (both tools); ICCs > 0.86; strong correlation (*r* ≈ 0.89); GOHAI better for functional discrimination	OHRQoL measurement in older adults; comparative evaluation; Arabic tool validation
Feng et al. (2022) [40]	Chinese	China	College students (*n* > 1,000)	Cross-sectional psychometric validation, including factor analysis and measurement invariance testing	Chinese OHIP-14	Rigorous translation; confirmatory factor analysis; tested invariance across genders	Cronbach’s α (total scale) = 0.96; subscale α ≈ 0.77–0.90; test–retest *r* ≈ 0.72; factorial validity and gender invariance supported	Large-sample psychometric validation; advanced statistical evaluation
Finotto et al. (2020) [41]	Italian	Italy	Inmates/patients aged ≥65 years with cognitive deficits (*n* = 368)	Linguistic-cultural validation using Beaton, Sousa, Rojjanasrirat models; face/content validity; test–retest reliability	OHAT (Italian version)	Forward/back translation; expert panel; pilot-tested with elderly; clarity assessment ≥80%; content validity: ICVI ≥0.8, SCVI = 0.93	Cronbach’s α = 0.82; test–retest Pearson’s *r* = 0.50; factor analysis supported tool structure	Validation of OHAT; elderly oral health assessment; cross-cultural tool adaptation
Gera et al. (2020) [29]	Danish	Denmark	Adults (orthodontic patients, *n* ≈ 200)	Translation and cross-cultural adaptation study	Danish OHIP-14	WHO forward/backward method; expert panel; cognitive interviews; tested comprehensibility	Cronbach’s α = 0.75–0.84, ICC = 0.91 factor analysis supported construct validity	Linguistic-cultural validation; Scandinavian population
Goje et al. (2017) [30]	Gujarati	India (Gujarat)	Adults (*n* = 109) from dental OPD	Cross-sectional translation and validation using WHO forward–backward method; bilingual administration in English and Gujarati	OHIP14 (Gujarati)	WHO standard forward/back translation; expert panel review; bilingual participants completed both language versions	Cronbach’s α = 0.98; Pearson’s *r* = 0.99 between versions; unpaired t-test non-significant (*P* = 0.103)	Language adaptation; cross-cultural tool validation
Khoshnevisan et al., (2016) [31]	Arabic	Iran/Iraq (Arabic-speakers)	Adults in general population (*n* ≈ 320 sample for reliability + 25 for test–retest)	Cross-sectional translation and validation study using standard forward/back translation, expert panel (NGT), pilot testing, and CFA	WHO Oral Health Assessment Questionnaire (Arabic version)	Forward/back translation; removed culturally inappropriate item; expert panel review; nominal group technique; pilot testing (*n*=50)	Cronbach’s α = 0.85 (subscales 0.75–0.91); ICC = 0.88; CFA: CFI = 0.89, RMSEA = 0.041; impact score ≥1.6; content validity index = 0.90, content validity ratio = 0.81	Cross-cultural validation; tool adaptation for Arabic-speaking adults; oral health surveillance instrument
Montero-Martín et al. (2009) [42]	Spanish	Spain (Granada)	Healthy working adults (*n* = 270)	Cross-sectional validation with pilot testing and clinical exams	OHIP14sp (Spanish version)	Linguistic-cultural adaptation; pilot tested with focus groups; self-administered; WHO dental exams conducted	Cronbach’s α = 0.89; strong criterion, construct, convergent validity; correlated with perceived need and caries (# teeth needing extraction *r* = 0.21, *p*<0.01)	Cross-cultural validation; OHRQoL assessment; tool adaptation
Murererehe et al. (2024) [32]	Kinyarwanda	Rwanda	Adults living with HIV and HIV-negative adults (*n* ≈ 400)	Comparative cross-sectional translation and validation study	OHIP-14 (Kinyarwanda version)	Forward–backward translation, expert panel review, pilot testing, and cultural adaptation for Rwandan context	Cronbach’s α > 0.85; test–retest ICC > 0.80; confirmed construct validity across HIV and non-HIV groups	Cross-cultural validation; application of OHIP-14 in African context; OHRQoL comparison in clinical vs. general populations
Motallebnejad et al. (2011) [43]	Persian	Iran	Adults aged 18–65 years (*n* = 325 dental clinic patients)	Cross-sectional translation and validation Study with pilot-testing and clinical assessment	OHIP14 (Persian version)	Forward/back translation, pilot-tested with 30 participants; face/content validation; clinical correlation	Cronbach’s α = 0.73–0.88 across domains; ICC = 0.82; significant correlations with clinical indices (e.g., DMFT *r* = 0.47, *p* < 0.01)	Cross-cultural validation; OHRQoL adaptation; clinical relevance
Papagiannopoulou et al. (2012) [33]	Greek	Greece (Athens, Thessaloniki)	Adults (*n* = 211), ≥35 years	Cross-sectional validation using forward/back translation; pilot-tested with 20 adults; clinical oral exams; interviewer-administered questionnaire	OHIP14 (Greek version)	Four independent forward and backward translations; cultural revisions; pilot testing; interviewer-administered	Cronbach’s α = 0.90; inter-item correlations = 0.10–0.83; item-total correlations = 0.44–0.76; significant associations with DMFT and oral hygiene; convergent validity with self-perceived oral health (rs = 0.57, *p* = 0.01)	Cross-cultural validation; OHRQoL measurement; instrument adaptation
Tefera et al. (2022) [7]	Amharic (basic translation only)	Ethiopia (Amhara region)	Hearing-impaired students in special need schools (*n* = 149; age 7–30 years)	Cross-sectional study using a pretested interview Questionnaire and clinical examination; used the WHO Oral Health Survey form	WHO-OHAF (translated)	Forward translation used; questionnaire pretested. Clinical exams included DMFT, CPI, and Simplified Oral Hygiene Index.	Prevalence data: 38.9% caries; 22.8% periodontal disease; logistic regression identified risk factors (AORs reported)	Oral health status assessment; initial use of Amharic WHO tool; gap in culturally validated OHRQoL instruments
Van der Meulen et al. (2008) [36]	Dutch	Netherlands	Adults from general population and clinical samples (*n* ≈ 200–300)	Cross-sectional translation and validation study	OHIP-NL (Dutch OHIP-14)	Forward/backward translation, expert review, pilot testing; evaluated construct validity and reliability	Cronbach’s α > 0.90 across domains; test–retest ICC > 0.85; strong construct validity with oral health indicators	Cross-cultural validation; European context; OHRQoL adaptation
Warsi et al. (2018) [34]	Urdu	Pakistan (Karachi)	Adults with upper gastrointestinal/hepatic disorders (*n*=350)	Cross-sectional validation incorporating clinical exams and Urdu OHIP-14 administration	OHIP14 (Urdu version)	Forward/back translation; pilot testing with 30 participants; interviewer-led survey aligned with clinical findings	Cronbach’s α = 0.92; ICC = 0.87; significant correlation with clinical oral findings and quality-of-life scores (*p* < 0.001)	OHRQoL tool validation; instrument adaptation for Urdu-speaking clinical population
Wąsacz et al. (2019) [45]	Polish	Poland (Wrocław region)	Adults with oral mucosa lesions or periodontal disease (*n* = 120)	Cross-sectional validation of a modified OHIP-14 In a clinical sample, a self-administered questionnaire and an oral exam	OHIP14 (Polish modified)	Forward/back translation; item modification for mucosal lesion relevance; pilot tested with 20 patients	Cronbach’s α = 0.91; test–retest reliability ICC = 0.88; strong construct validity (*r* = 0.65–0.79 with oral clinical indices)	Clinical tool adaptation; instrument modification for specific oral pathologies
Yamazaki et al. (2007) [35]	Japanese	Japan	Adults (*n* = 202) from community dental clinics	Cross-sectional validation study using a self-administered questionnaire and structured interviews	OHIPJ (Japanese OHIP14)	Rigorous translation/back-translation; cultural adaptation of colloquialisms; Pilot tested with 30 participants	Cronbach’s α = 0.92; test–retest ICC = 0.90; Factor analysis supported a 7-domain structure. strong construct validity (*r* = 0.76)**	OHRQoL tool adaptation; instrument validation in the Japanese community

Montero-Martín et al. (2009) [42] used the OHIP-49 (full version) rather than the OHIP-14. This study was included due to its methodological relevance and contribution to Spanish-language adaptation and validation processes aligned with OHIP-14

*Abbreviations: α* Cronbach’s alpha, *A-OHAT* Arabic-WHO Oral Health Assessment Tool, *AOR* Adjusted Odds Ratio, *CFA* Confirmatory Factor Analysis, *CFI* Comparative Fit Index, *CPI* Community Periodontal Index, *DMFT* Decayed, Missing, and Filled Teeth index, *EFA* Exploratory Factor Analysis, *GOHAI* Geriatric Oral Health Assessment Index, *ICC* Intraclass Correlation Coefficient, *ICVI* Item Content Validity Index, *I-CVI* Item-level Content Validity Index, *OHAT* Oral Health Assessment Tool, *OHIP* Oral Health Impact Profile, *OHRQoL* Oral Health-Related Quality of Life, *RMSEA* Root Mean Square Error of Approximation, *r* Pearson correlation coefficient, *rs* Spearman’s rank correlation coefficient, *S-CVI* Scale-level Content Validity Index, *S-CVI/Ave* Average Scale-level Content Validity Index, *t* t-test, *WHO* World Health Organisation

Cronbach’s α values typically exceeded 0.70, with several studies reporting values above 0.90, indicating strong internal consistency [26–47]. Test–retest reliability was also frequently assessed using ICCs, which demonstrated good to excellent stability over time. Validity was supported through exploratory and confirmatory factor analyses, as well as through associations with clinical indicators and self-rated oral health. WHO-OHAFT studies reported good utility for assessing dental caries, periodontal status, and prosthetic needs.

Table 4 summarises internal consistency and test–retest reliability values and indicate variability in how cross-cultural adaptation procedures were reported.

**Table 4:** Psychometric testing and cross-cultural adaptation details of included studies

Author(s) and Year	Cronbach’s α	Test-Retest (ICC)	Content Validity	Factor Analysis	Cross-Cultural Protocol Followed
Al Habashneh et al. (2012) [37]	✓ 0.89	**X**	✓	**X**	✓ Partial
Bae et al. (2007) [26]	✓ 0.97	✓ 0.64	✓	**X**	✓ Beaton
Balcı et al. (2017) [27]	✓ 0.76–0.91	✓ 0.89	✓	**X**	✓ Partial
Bokhari & Quadri (2020) [38]	✓ 0.72	✓ 0.89	✓	**X**	✓ Partial/Other
Couto et al. (2018) [28]	✓ 0.92	✓ 0.999	✓	✓	✓ Beaton
Deshpande & Nawathe (2015) [46]	✓ 0.80	**X**	✓	**X**	✓ Partial
El Osta et al. (2012) [39]	✓ 0.91	✓ 0.86	✓	**X**	**X**
Feng et al. (2022) [40]	✓ 0.96	**X**	✓	✓ (CFA, invariance)	**X**
Finotto et al. (2020) [41]	✓ 0.82	**X**	✓	✓	✓ Beaton & Sousa
Gera et al. (2020) [29]	✓ 0.75–0.84	✓ 0.91	✓	**X**	✓ Beaton
Goje et al. (2017) [30]	✓ 0.98	**X**	✓	**X**	✓ Partial
Khoshnevisan et al., (2016) [31]	✓ 0.85	✓ 0.88	✓	✓ (CFA)	✓ Beaton
Montero-Martín et al. (2009) [42]	✓ 0.89	**X**	✓	✓	✓ Beaton
Motallebnejad et al. (2011) [43]	✓ 0.73–0.88	✓ 0.82	✓	**X**	✓ Beaton
Murererehe et al. (2024) [32]	✓ >0.85	✓ >0.80	✓	✓	✓ Beaton
Papagiannopoulou et al. (2012) [33]	✓ 0.90	**X**	✓	**X**	✓ Beaton
Tefera et al. (2022) [7]	**X**	**X**	**X**	**X**	**X** (basic translation only)
Van der Meulen et al. (2008) [36]	✓ >0.90	✓ >0.85	✓	✓	✓ WHO method
Warsi et al. (2018) [34]	✓ 0.83	**X**	✓	**X**	✓ Guillemin-style translation
Wąsacz et al. (2019) [45]	✓ 0.91	✓ 0.88	✓	✓	✓
Yamazaki et al. (2007) [35]	✓ 0.92	✓ 0.90	✓	✓	✓ Beaton

✓ = Reported; X = Not Reported

*ICC* Intraclass Correlation Coefficient, *CFA* Confirmatory Factor Analysis

### Thematic findings

Table 5 illustrates the summary of the themes that emerged from the thematic analysis.

**Table 5:** Inductively derived themes

Themes	Descriptions
Application of OHIP-14 and WHO-OHAF	Use of these tools across varied cultural and linguistic settings.
Adaptation and Validation Practices	Methods such as translation fidelity, expert consultation, and psychometric testing.
Gaps in Amharic Tools	Lack of validated Amharic-language instruments for subjective and clinical oral health assessment.

#### Theme 1: application of OHIP-14 and WHO-OHAFT

This theme addresses how OHIP-14 and WHO-OHAFT have been applied across diverse ethnolinguistic and culturally diverse populations, including their capacity to capture subjective OHRQoL outcomes. The OHIP-14 was the most widely applied tool across studies, adapted for diverse cultural and linguistic settings to measure subjective OHRQoL outcomes [12, 26, 33, 34, 39, 42]. Applications included both clinical populations and community-based surveys. In contrast, the WHO-OHAFT and its derivatives (e.g., OHAT, A-OHAT) were primarily used for objective clinical assessments, focusing on indicators such as caries, periodontal status, and functional impairments [1, 7, 15, 38, 41]. OHIP-14 captured psychosocial dimensions of oral health, whereas WHO-OHAFT focused on objective clinical indicators, as reported in studies [7, 38, 39].

#### Theme 2: adaptation and validation practices

Approaches to adaptation and validation varied considerably. Some studies reported using multi-step adaptation procedures that included forward–backward translation, expert panel review, pilot testing, and psychometric evaluation [28, 31]. For example, the Portuguese OHIP-14 reported Cronbach’s α = 0.92 and ICC = 0.99 [28], while the Arabic WHO tool used in Iran/Iraq reported Cronbach’s α = 0.85, with CFA (Confirmatory Factor Analysis) results supporting validity [31]. Other studies provided minimal detail regarding their adaptation procedures, including the Hindi OHIP-14 [46] and the Amharic translation of the WHO-OHAFT [7].

Reported psychometric values ranged from Cronbach’s α = 0.73 in the Persian OHIP-14 [43] to α = 0.97 in the Korean OHIP-K [26]. Test–retest reliability was frequently reported, with ICCs often exceeding 0.80 across several studies [35]. Only a subset of studies reported cognitive testing or participant involvement in the adaptation process [7, 28, 46], indicating that the reporting of adaptation procedures varied across studies.

#### Theme 3: gaps in amharic tools

Across the included studies, validated Amharic-language tools for assessing OHRQoL were largely absent. Across the literature, only one study addressed an Amharic-language adaptation of WHO-OHAFT. This study provided a basic Amharic translation intended for preliminary use rather than as part of a full validation process [7]. No psychometric evaluation of an Amharic-language instrument was reported.

## Discussion

This hybrid systematic–narrative review synthesised evidence from 21 studies evaluating the cross-cultural adaptation and validation of the OHIP-14 and WHO oral health assessment tools across diverse linguistic and cultural settings [7, 26–47]. Although the primary aim was to determine whether validated Amharic-language tools exist for refugee use, the available evidence base was almost entirely non-Amharic. The review, therefore, deliberately engages with multilingual and cross-cultural adaptation studies across 21 languages to derive transferable methodological insights for future Amharic-language tool development in refugee and migrant contexts. Three key themes were identified: [1] application of OHIP-14 and WHO-OHAFT, [2] adaptation and validation practices, and [3] the absence of validated Amharic-language tools. Overall, many studies showed strong psychometric performance and followed recognised cross-cultural adaptation procedures, providing confidence in the global applicability of these tools [26–28, 40]. However, limited reporting in some studies raises concerns about cultural validity and comparability [7, 46]. Notably, no validated Amharic-language version of these instruments has been reported, despite the growing need to capture oral health outcomes in Ethiopian refugee populations. This omission underscores a broader gap in linguistic inclusivity within oral health research [4, 21, 49]. Because only one included study involved an Amharic-speaking population, evidence from wider multilingual contexts was essential for drawing methodological insights relevant to Amharic adaptation. These findings directly address both research questions by illustrating how OHIP-14 and WHO-OHAFT have been applied and adapted across cultural settings, while demonstrating the absence of a validated Amharic-language tool. This interpretation aligns with the hybrid systematic–narrative design, and with equity-oriented frameworks that emphasise culturally and linguistically grounded measurement in marginalised populations [50, 51].

### Application of OHIP-14 and WHO-OHAFT

The consistent psychometric reliability of OHIP-14 across populations suggests it could be successfully adapted for refugee settings [26–43, 45, 46], yet its underuse in these contexts indicates a missed opportunity to capture patient experiences [5, 21, 52]. Conversely, the continued reliance on WHO-OHAFT as a clinical screening tool [14, 16] reinforces its limited value for assessing lived experiences in displaced communities [4, 5, 21, 52]. Across the 21 included studies Cronbach’s α values ranged from 0.72 to 0.98, with a mean of 0.88, indicating consistently strong internal consistency across most linguistic adaptations of OHIP-14 and WHO-OHAFT [26–46]. ICCs were reported in 15 of the 21 studies, with values ranging from 0.64 to 0.999 and exceeding 0.80 in most cases, supporting robust test–retest reliability [26–28, 31, 33–37, 39, 42–46]. Regional patterns were observed: Arabic- and Portuguese-language adaptations (e.g., Couto et al., Khoshnevisan et al.) generally reported high α (≥ 0.85) and ICCs ≥ 0.88, reflecting strong psychometric performance [28, 31, 37, 39, 44]. Asian-language versions (e.g., Korean, Japanese, Chinese) also demonstrated strong metrics, though some (e.g., Korean) had slightly lower test–retest ICCs (e.g., 0.64) [26, 35, 40]. These findings confirm that, when rigorous adaptation protocols (e.g., Beaton or WHO methods) are followed, both OHIP-14 and WHO-OHAFT can yield high reliability across culturally and linguistically diverse populations [14, 16]. These patterns support the potential suitability of both tools for culturally and linguistically diverse populations, including refugee groups, provided that rigorous cross-cultural adaptation is undertaken.

Tool adaptation requires both methodological rigour and cultural sensitivity. Frameworks such as Beaton et al. [14] and Sousa & Rojjanasrirat [16] provide structured approaches to ensure conceptual and linguistic equivalence [53, 54]. Adaptations in Arabic, Greek, and Japanese [33, 35, 39] demonstrate how cultural beliefs about pain, hygiene, and emotional expression must inform item revision. These practices also apply to WHO-OHAFT and OHAT, where successful versions incorporated culturally specific constructs to improve validity [31, 41]. However, not all studies adhered to these rigorous procedures, and in some cases, key adaptation steps were omitted, raising concerns about validity and comparability. Studies omitting key adaptation steps (e.g., back-translation, cognitive interviews) risk reduced validity and hinder cross-study comparability [14, 16, 55]. Adherence to frameworks [14, 16] is essential for reliability and future meta-analyses. Detailed reporting of adaptation processes should be standard to enhance transparency.

### Gaps in amharic tools

The absence of a validated Amharic-language version of OHIP-14 or WHO-OHAFT has significant implications for oral-health assessment among Amharic-speaking Ethiopian refugees. Without culturally and linguistically adapted tools, assessments risk misrepresentation of symptoms and raise ethical concerns related to informed consent, measurement validity, and fairness, particularly given the cultural beliefs and practices that shape oral-health perceptions in Ethiopian communities [7, 14, 16, 56]. Standardised oral health instruments often fail to account for the cultural and psychosocial contexts of displaced populations [4, 8, 16, 56]. For Amharic-speaking Ethiopian refugees, oral health beliefs may include traditional remedies, communal decision-making, and spiritual interpretations of pain, elements not captured by standard tools [8, 56]. Similar challenges have been observed among Syrian refugees in Aotearoa, where dual-language resources led to improved oral health behaviours but structural barriers, including cost and lack of cultural safety, persisted [5, 16, 57]. Although OHIP-14 has been used in refugee populations, such as in studies by Fink et al. (2024) and Ziersch et al. (2025), these applications lacked cultural adaptation [21, 52], increasing the risk of underreporting pain, overlooking traditional oral practices, or misrepresenting psychosocial impacts. This gap reflects a broader inequity in global oral-health measurement, where the development and validation of tools have rarely extended to marginalised or refugee populations [4, 21, 49]. Evidence from adjacent health fields reinforces the importance of cultural adaptation. Culturally adapted mental-health tools for refugee populations, such as those described by Gadeberg et al. (2017) [53] and Kasujja et al. (2022) [54], demonstrate that rigorous adaptation is both feasible and essential. The oral-health field must follow suit by prioritising community-informed, culturally responsive tool development for Ethiopian and other underrepresented refugee populations.

The cultural adaptation and validation of oral health instruments must be understood not only as a methodological exercise but also as an act of equity. Frameworks such as the WHO Health Equity Framework [50] and Browne et al.’s model of equity-oriented health care [51] emphasise the need for culturally safe, linguistically accessible, and community-informed health interventions. When applied to oral health, this means ensuring that tools like OHIP-14 and WHO-OHAFT reflect the values, experiences, and health beliefs of marginalised groups, including refugees [1, 3, 13, 58]. The lack of validated Amharic-language tools reflects a broader inequity in measurement science, where the perspectives of displaced and linguistically minoritised communities remain underrepresented [4, 49, 53, 54]. Cross-cultural adaptation, when conducted thoroughly and inclusively, becomes a mechanism for advancing epistemic justice and refugee health equity [14, 16, 50, 51, 55]. This approach aligns with global movements to decolonise health research and to ensure that no communities are excluded from culturally valid assessment, equitable care, or informed policy-making [1, 3, 51].

### Refugee-specific implications

Refugee oral-health experiences are shaped by displacement, resettlement pressures, trauma exposure, disrupted dental-care pathways, financial constraints, and difficulties navigating unfamiliar health systems, all of which increase vulnerability to poor oral health [4, 5, 21, 49, 58]. If OHRQoL instruments are not culturally adapted, these realities may be underrepresented in clinical assessments and population datasets, resulting in incomplete or misleading interpretations [14, 16, 24].

For Amharic-speaking Ethiopian refugees, cultural understandings of oral health, including traditional cleansing methods (e.g., mefakia), herbal remedies, communal care practices, and spiritual interpretations of pain, may influence how symptoms are perceived and expressed [7, 8]. Standard instruments may therefore fail to capture culturally grounded meanings of discomfort, disability, or social impact [14, 16]. This mismatch risks obscuring psychosocial burdens, such as stigma, reduced confidence, or disrupted cultural practices, which are particularly relevant in displacement contexts [4, 13, 52].

Accurately reflecting these culturally shaped experiences is essential for health-system responsiveness [1, 50, 51]. Culturally validated tools allow clinicians and policymakers to better identify unmet needs, tailor interventions, and design equitable oral-health programmes that align with the lived realities of refugee communities [1, 3, 51]. Without culturally adapted and validated instruments, oral-health needs among refugee populations may be systematically underestimated, limiting the quality of evidence used to shape equitable service planning and policy development [5, 14, 16, 21].

### Strengths and limitations

A key strength of this review is its innovation, as to the authors’ knowledge, it represents the first systematic effort to document the adaptation and validation of OHIP-14 and WHO oral health assessment tools across diverse cultural and linguistic contexts, with a particular focus on Amharic-speaking and refugee populations. The use of a hybrid systematic–narrative approach [17] enabled both structured mapping and in-depth thematic interpretation [25], allowing for a nuanced synthesis of methodological practices and psychometric outcomes. By drawing evidence from multiple countries and languages, the review offers a broad cross-cultural perspective and provides a methodological foundation relevant for future Amharic-language adaptations, which are currently lacking.

Nonetheless, several limitations should be acknowledged. Restricting the review to peer-reviewed literature in English introduces potential publication and language bias [18, 23]. Studies varied in quality, sample size, and outcome reporting, limiting synthesis consistency [18, 59, 60]. No included validation studies directly sampled refugee populations, which limits the direct generalisability of these validation findings to refugee settings [4, 21]. While the hybrid systematic–narrative approach enabled both mapping and interpretations [18, 19, 22] it may also introduce subjectivity despite predefined coding domains. Heterogeneity in study methods and reported outcomes (e.g., internal consistency versus construct validity) also complicates the comparison across settings [4, 58, 61]. Finally, the search strategy may have excluded non-English validation studies (e.g., Farsi, Arabic, Hindi) due to database and language constraints [59, 60].

### Future implications

Future efforts should prioritise strengthening culturally appropriate oral health assessment in refugee and migrant contexts [1, 3, 4]. Greater collaboration with Amharic-speaking communities is essential to ensure that adapted instruments reflect lived experiences and cultural perspectives [2]. Integrating validated tools into refugee oral-health pathways, such as initial health assessments, community dental services, and culturally tailored health-promotion programmes, could improve screening, support targeted health promotion, and strengthen evidence-based policy [1–4]. Building capacity among Amharic-speaking oral health professionals in migrant-focused services would further support equitable delivery of care [51, 58]. Developing validated Amharic tools is a policy imperative: without them, refugee oral health programmes risk relying on culturally invalid data, limiting both health promotion and equitable policy development [14, 16]. Embedding these instruments into routine oral health services is therefore critical for addressing disparities and ensuring that policy decisions are grounded in culturally valid data [2, 3, 21, 49]. Clear implementation pathways will be needed so that these tools are incorporated into clinical practice, integrated into workforce training, and recognised within national oral-health equity frameworks [1–3].

Future research should extend beyond existing work by focusing on the translation and validation of OHIP-14 and WHO-OHAFT in Amharic [8, 14, 16, 55], directly addressing the current absence of robust tools for this population. Research should also broaden adaptation studies to underrepresented refugee groups [21, 53, 62] and improve transparency in adaptation methodologies by clearly reporting procedures such as translation steps, cultural adaptation decisions, and psychometric testing [14, 22, 55]. In addition, incorporating participatory approaches and community involvement throughout the adaptation and validation process will help ensure cultural resonance and strengthen trust in oral-health assessment tools [50, 51, 58].

## Conclusion

This hybrid systematic–narrative review highlights the absence of validated Amharic-language OHRQoL instruments reported in the literature, which may limit accurate assessment and equitable care for Amharic-speaking and Ethiopian-origin populations. Addressing this gap requires the rigorous translation, cultural adaptation, and participatory validation of existing tools such as the OHIP-14 and WHO-OHAFT. Integrating adapted instruments into clinical and public health systems is vital to ensure linguistic and cultural relevance within oral health assessment in research and practice. Advancing linguistic inclusion in tool development and deployment is a critical step toward reducing global oral health inequities and ensuring that refugee and migrant populations are equitably represented in research, care, and policy. Ultimately, developing and implementing validated Amharic-language oral-health tools is an essential foundation for improving assessment accuracy, strengthening health-system responsiveness, and advancing oral-health equity for Amharic-speaking refugee-displaced populations.

## Data Availability

All data generated or analysed during this review are included in this published article and its submitted tables and figures. Coding spreadsheets and search logs are available from the corresponding author upon reasonable request.

## Abbreviations

OHIP-14: Oral Health Impact Profile – 14-item version
OHRQoL: Oral Health-Related Quality of Life
WHO: World Health Organisation
OHAT: Oral Health Assessment Tool
GOHAI: Geriatric Oral Health Assessment Index
PRISMA: Preferred Reporting Items for Systematic Reviews and Meta-Analyses
ICC: Intraclass Correlation Coefficient
EFA: Exploratory Factor Analysis
CFA: Confirmatory Factor Analysis
IRT: Item Response Theory
DIF: Differential Item Functioning
CVI: Content Validity Index
CVR: Content Validity Ratio
DMFT: Decayed, Missing, and Filled Teeth Index
CPI: Community Periodontal Index
A-OHAT: Arabic Oral Health Assessment Tool
ADD: Additive score
SC: Simple Count score
NGT: Nominal Group Technique
S-CVI: Scale-level Content Validity Index
I-CVI: Item-level Content Validity Index
FDI: World Dental Federation
NZ: New Zealand
